# Trauma in elderly people: access to the health system through pre-hospital care^1^

**DOI:** 10.1590/1518-8345.0959.2690

**Published:** 2016-05-03

**Authors:** Hilderjane Carla da Silva, Renata de Lima Pessoa, Rejane Maria Paiva de Menezes

**Affiliations:** 2 MSc, RN, Pronto Socorro Clóvis Sarinho, Hospital Monsenhor Walfredo Gurgel, Natal, RN, Brazil; 3 MSc, RN, Serviço de Atendimento Móvel de Urgência, Natal, RN, Brazil; 4 PhD, Associate Professor, Departamento de Enfermagem, Universidade Federal do Rio Grande do Norte, Natal, RN, Brazil

**Keywords:** Nursing, Pre-Hospital Care, Wounds and Injuries, Elderly

## Abstract

**Objective:**

to identify the prevalence of trauma in elderly people and how they accessed the health system through pre-hospital care.

**Method:**

documentary and retrospective study at a mobile emergency care service, using a sample of 400 elderly trauma victims selected through systematic random sampling. A form validated by experts was used to collect the data. Descriptive statistical analysis was applied. The chi-square test was used to analyze the association between the variables.

**Results:**

Trauma was predominant among women (52.25%) and in the age range between 60 and 69 years (38.25%), average age 74.19 years (standard deviation±10.25). Among the mechanisms, falls (56.75%) and traffic accidents (31.25%) stood out, showing a significant relation with the pre-hospital care services (p<0.001). Circulation, airway opening, cervical control and immobilization actions were the most frequent and Basic Life Support Services (87.8%) were the most used, with trauma referral hospitals as the main destination (56.7%).

**Conclusion:**

trauma prevailed among women, victims of falls, who received pre-hospital care through basic life support services and actions and were transported to the trauma referral hospital. It is important to reorganize pre-hospital care, avoiding overcrowded hospitals and delivering better care to elderly trauma victims.

## Introduction

In recent decades, Brazil, like other countries in Latin America, observed technological innovations in the health sector. Nevertheless, the country continues struggling for universal coverage, as the epidemiological changes deriving from the aging of the population generated new demands for care delivery to non-transmissible diseases, among which the chronic-degenerative diseases, tumors and traumas stand out^(^ 1 ^)^.

Although trauma rates among young people are high, this kind of events rank fifth in terms of global mortality in the elderly population, which is affected as a result of exposure to risk factors like the reduction of the physiological reserves and functional capacity, related to the frailty syndrome, comorbidities, alcohol consumption, multiple medication, inappropriate structure of spaces, traffic dynamics and inclusion in the job market^(^ 2 ^-^ 3 ^)^.

The peculiarities of elderly people, such as the degree of frailty, chances of infections and bleeding, hemodynamic instability, greater feeling of pain and presence of comorbidities commonly require specific and even intensive care. These circumstances generate new demands in health care, contribute to the increase of care service spending, hospitalizations, institutionalizations, morbidity and mortality, resulting in a social and economic burden^(^ 4 ^-^ 8 ^)^.

In view of the increase in trauma events among elderly people and the importance of immediate care to define a good prognosis, considering that trauma is time-dependent, Mobile Pre-Hospital Care (MPHC) is relevant, as this care form is delivered by a medical and nursing team that provides initial care while still at the event site, through mobile services like Basic Life-Support (BLS), Advanced Life-Support (ALS) and Motorcycle Ambulances^(^ 3 ^,^ 9 ^-^ 10 ^)^.

MPHC works in accordance with the Brazilian Emergency Care Services and the premises of the Unified Health System (SUS), including the principles of universality, equity and integrality and the guidelines of decentralization, prioritization and regionalization, as it operates through a Regulation Central that permits organizing the services to prevent overcrowded hospitals and emergency care services^(^ 11 ^)^.

The difficulty to obtain hospital beds, however, is a generalized problem in the health system and is further exacerbated with regard to elderly patients, due to the more specific care required^(^ 12 ^)^. The lack of an organized health network makes the care services inefficient to respond to the demand in view of the progressive population aging, hampering the elderly population's access to high-quality services and leading to a loss of resource efficiency, consequently increasing costs in the health area^(^ 13 ^)^.

Hence, the growth of the elderly population, in combination with the demand trauma events pose, represents new challenges for the emergency services, especially in the pre-hospital care context, as a result of elderly people's particularities, which make them more vulnerable to trauma events and their consequences, with the necessary access to a universal and high-quality health system that has been structured to respond to this reality^(^ 14 ^)^.

As an emergent health problem, the understanding of trauma in the elderly population enables the multiprofessional team, which nursing is part of, to plan and implement strategies for more specific geriatric care and, hence, to contribute to the reduction of the chances of temporary or permanent sequelae, as well as to the prevention of these events, with a view to balancing the health system as a whole.

In emergency health services, nursing plays a fundamental role to achieve universal access to these services by applying its knowledge and fundamentals in the use of critical and reflexive reasoning on this care, considering its presence at the different complexity levels of health care^(^ 15 ^)^. MPHC is part of the care modalities not only in terms of care, but also in management.

To analyze the problem, in this study, the following question was raised: what is the prevalence of trauma in elderly people and how do they access the health system through MPHC?

The objective was to identify the prevalence of trauma in elderly people and how this population gets access to the health system through MPHC. It is relevant because studies on the magnitude, dynamics and understanding of trauma in elderly people are increasingly necessary in the health area, as they permit broadening the knowledge on this problem, with strong economic and social impacts. It should also be highlighted that the expansion of knowledge permits the enhancement of strategies to improve health service care.

## Method

Descriptive and cross-sectional study with a retrospective documentary design, based on secondary data sources in the Mobile Emergency Care Service of the city of Natal, Rio Grande do Norte (SAMU/Natal-RN), Brazil.

Records on trauma events in elderly people attended between January 2011 and December 2012 were analyzed, totaling a population of 2,080 elderly trauma victims, corresponding to 8.46% of all trauma events the service attended in the same period.

The data were surveyed between July and September 2013. The sample size was estimated as follows: n_0_=Z^2^P^2^/E^2^_0_, with n indicating the sample size, Z the 95% confidence interval (Z=1.96), P the probability of finding the study phenomenon (P=0.5) and E the tolerated sampling error (E=0.05). Thus, a minimum sample size of 384 trauma victims was obtained, which was rounded off to 400 to minimize the risks of sampling error.

The sample was selected through systematic random sampling, whose first element was drawn and the remainder followed the interval K=N/n, in which N corresponded to the population (N=2080) and n to the sample (n=400), revealing the constant K=5.2, rounded off to 5. Hence, for every five trauma events in elderly people, the fifth was selected for the study sample.

The following eligibility criteria were adopted: age 60 years or older, trauma victims, attended between January 2011 and December 2012. Records that were illegible were excluded, which made it difficult to reach the study objective. The variables considered in this research excerpt were: sex and age of the victims, trauma mechanism, care service, team actions and destination of the victim.

To collect the data, a form was used the researchers had elaborated, which was validated by expert judges, for the sake of retrospective analysis of the care records, which the nursing team from the service had completed during the selected period.

The collected data were registered in Microsoft Excel 2010 and imported to Statistical Package for Social Science (SPSS), version 20.0. To verify the association among the variables, Pearson's chi-square test was applied (X²) with a 95% confidence interval and a 5% significance level 5% (p<0.005).

The study was developed with the preliminary consent of the coordination of SAMU/Natal-RN and a favorable opinion from the Research Ethics Committee at Universidade Federal do Rio Grande do Norte (CEP/UFRN), under Protocol 309.505/2013.

## Results

Trauma cases mainly affected women between 60 and 69 years of age, as shown in Table 1. The mean age was 74.19 years, with a standard deviation (sd) of 10.25 (sd±10.25).

**Table 1:** - Distribution of trauma events in elderly according to sex and age range. Natal, RN, Brazil, 2013

**Age range**	**Sex**				**Total**	
	**Female**		**Male**			
	**n**	**%**	**n**	**%**	**n**	**%**
60|-|69 years	54	25.9	99	51.9	153	38.25
70|-|79 years	68	32.5	52	27.2	120	30
80|-|89 years	57	27.3	35	18.3	92	23
90|-|99 years	26	12.4	4	2.1	30	7.5
100|- years	4	1.9	1	0.5	5	1.25
Total	209	100	191	100	400	100
%	52.25		47.75		100	

The results displayed in Table 2 evidence that the BLS are the most used services for care delivery to elderly trauma victims, corresponding to more than 85% and present in care delivery to the different trauma mechanisms, despite being more represented in cases of falls, the prevalent mechanism in elderly people.

**Table 2:** - Distribution of trauma events in elderly victims according to trauma mechanism and care service. Natal, RN, Brazil, 2013

**Trauma mechanism**	**Care service**					**Total**
	BLS*	**ALS†**	**BLS+** **ALS**	**BLS+** **Motor‡**	**ALS+BLS+** **Motor**	
Falls	212	2	8	5	0	227
	93.4%	0.9%	3.5%	2.2%	0%	100%
Traffic accidents	104	5	13	1	2	125
	83.2%	4.0%	10.4%	0.8%	1.6%	100%
Violence	6	2	4	1	0	13
	46.2%	15.4%	30.8%	7.7%	0%	100%
Burns	5	0	0	0	0	5
	100%	0%	0%	0%	0%	100%
Others**§**	24	2	4	0	0	30
	80%	6.7%	13.3%	0%	0%	100%
Total	351	11	29	7	2	400
	87.8%	2.8%	7.2%	1.8%	0.5%	100%
p value||	<0.001					

*BLS=Basic Life Support; †ALS=Advanced Life Support; ‡Motor**=**Motor Ambulance; §Others=poisoning, airway obstruction by strange body and collision with fixed objects; ║p value=obtained through chi-square test

ALS were the most used services in traffic accidents, in some cases associated with BLS, as shown in Table 2. The X^2^ test evidenced a significant association between the trauma mechanism and the care service.

As regards the actions performed by or in cooperation with the nursing team in MPHC, circulatory actions were prevalent, mainly volemic replacement, as well as limb immobilization using the rigid board and airway opening with cervical control. These data are summarized in Table 3, as follows.

**Table 3:** - Distribution of actions by or in cooperation with nursing team in MPHC. Natal, RN, Brazil, 2013

**Actions**	**n**	**%**	
Airway opening and cervical control			
	Placement of cervical collar	181	45.25
	Endotracheal intubation	07	1.75
	Total	188	47
Ventilation			
	Use of bag-valve-mask	08	2
	Oxygen therapy (oxygen mask/nasal catheter)	34	8.5
	Total	43	10.75
Circulation			
	Compressive dressing	72	18
	Peripheral venous access	78	19.50
	Volemic replacement	96	23.25
	Cardiopulmonary reanimation	05	1.25
	Total	251	62.75
Neurological dysfunction assessment			
	Glasgow coma scale	148	37
	Total	148	37
Immobilization			
	Limb immobilization	50	12.25
	Use of rigid board	138	34.50
	Total	188	47
Destination			
	Referral trauma hospital	227	56.7
	Public hospitals	77	19.2
	Private hospitals	61	15.3
	Kept in same place	35	8.8
	Total	400	100.0

As for the victim's destination, after the mobile pre-hospital care, a clear concentration of transports to the public hospitals was evidenced, to the detriment of the private network, as shown in Table 3. The referral hospital received more than half of the elderly trauma victims attended by SAMU/Natal-RN.

Table 3 also reveals that almost 10% of the trauma victims were kept at the place of the event. This fact was due to different causes, among which deaths, refusal of care or transportation by the victim and/or relatives stood out, as well as removal not indicated by the physician attending the event. In these cases, the MPHC team provides orientations on the necessary conducts to minimize possible sequelae.

## Discussion

The results achieved in this study in terms of the prevalence of trauma, sex, age and mechanism partially converge with the reports in the literature. In a similar study at an emergency service, it was evidenced that the elderly victims' mean age was 72.6 years, with sd±9.3 years, close to the present findings^(^ 10 ^)^.

The elderly's profile differs from the young population, as they are more affected by falls, traffic accidents (mainly collisions), burns and violence, as identified in this study and also appointed in the literature^(^ 4 ^,^ 9 ^-^ 10 ^)^.

The prevalence of the mechanism differs according to sex and age, as women over 69 years of age are the main victims of falls, due to the higher degree of frailty associated with osteoporosis, as a result of the post-menopause period, while men are more exposed to traffic accidents and in the age group between 60 and 69 years, due to the exposure to risk factors like greater circulation in the urban perimeter, leisure activities and the job market^(^ 4 ^-^ 5 ^,^ 16 ^-^ 17 ^)^.

In view of the magnitude and the impact the trauma can provoke in the morbidity and mortality rates among the elderly, MPHC, represented by the SAMU in Brazil is relevant because it not only provides early care, but also permits the organization of the hospital services the victims are destined to through the Regulation Central, according to the premises of the Brazilian Emergency Care Policy and the care decentralization concept, according to the complexity levels the Unified Health System puts forward^(^ 11 ^)^.

In the Regulation Central, the regulatory physician guides the citizen who summons the service and assesses the need to send the care resource and which is the most appropriate service in view of the severity of the situation^(^ 11 ^)^. In this study, it was evidenced that the BLS service delivered most of the care, pointing towards less severe injuries, linked to the prevalent trauma mechanisms which were falls, as well as the fact that, in many cases, ALS is summoned after the BLS team assessed the victim. These data converge with other similar results, in which BLS represents more than 80% of the pre-hospital care provided^(^ 4 ^,^ 10 ^,^ 18 ^)^.

The motor ambulances, as rapid intervention vehicles, are used to overcome obstacles related to intense traffic in the urban area and to get access to borderline areas^(^ 11 ^)^. Thus, they guarantee support for the transportation services and are commonly associated with BLS and/or ALS, justifying their lower care numbers, as identified in this study. In addition, it should be highlighted that these vehicles do not circulate at night, due to the lesser visibility, exposure to violence, reduction of traffic jams and the lesser number of events.

Besides the transportation, MPHC delivers the first care at the event site with a view to stabilizing the victim before removal. Concerning the initial care actions for the victim, it was observed that circulatory control, airway opening and cervical control, as well as immobilization were the actions most performed by or in cooperation with the nursing team in MPHC. These results are in line with the study in which it was appointed that airway opening, ventilation and circulatory control were performed in more than 92% of the events^(^ 19 ^)^.

The pre-hospital actions are fundamental to reduce the risks of death within the so-called golden hour, but as important as these actions is to transport the victim to a hospital for definitive treatment, considering the type of care needed, the location of the event, presence of geographical barriers, distance to the hospital and healthcare network infrastructure^(^ 18 ^)^.

In this study, the main concentration of victims to one specific hospital was observed, to the detriment of others, as well as the prevalence of public services as the final destination, as recommended by the regulatory policies of MPHC^(^ 11 ^)^ and the Regulation Central. This service aims to reduce the overcrowding of hospitals, but care through the referral hospital is based on the "zero vacancy" principle, as it has to be delivered independently of the existence of vacant beds, since emergencies demands fast and appropriate reactions^(^ 11 ^,^ 20 ^)^ and, in terms of universal health coverage, guarantees equity and the right to health^(^ 21 ^)^.

Authors refer that the main concentration of mobile care services to referral hospitals is in line with the hospital-centered care model and ignores the medical regulation protocols^(^ 18 ^)^. Others appoint the need for better organization and enhancement of MPHC in terms of regulation forms, in order to avoid the burden of the health services that provide definitive care^(^ 22 ^)^.

Although the zero vacancy policy is important, the overcrowding of hospitals should be taken into account, due to the difficulty to get beds immediately, especially for an elderly patient, who commonly requires specific and intensive care due to age and comorbidities, which can exacerbate the sequelae of the trauma event. In addition, after being hospitalized, the elderly's length of stay is longer, as well as the complications, increasing the costs of care.

As the nursing team participates in all care spheres in MPHC, it needs to deliver specific elderly care actions, contributing to the reduction of sequelae, as emergency care is one of the main factors defining the trauma victim's prognosis. Nevertheless, the importance of planning strategies to prevent trauma events should be highlighted.

This study is limited by the quality of the records, which made it impossible to assess the severity rates of the victims and the response time the MPHC used, as well as by the incipient nature of earlier studies at the same service with a view to comparing the results for better discussion. The method employed made it impossible to identify the causes of trauma in the elderly and their outcomes after the patients' hospital care.

These study findings can contribute to fundamental information for the reorganization of mobile pre-hospital care to elderly trauma victims, besides promoting reflection on the theme which today is a health problem around the world, as it causes strong impacts and interferes directly in the challenges to achieve appropriate universal health coverage.

## Conclusion

This study revealed the prevalence of traumas in elderly victims, mostly women between 60 and 69 years of age. This prevalence was mainly caused by falls, traffic accidents and violence, leading to the predominance of BLS in emergency care delivery by the mobile pre-hospital care service.

Concerning the actions taken by or with the cooperation of the nursing team in MPHC, circulatory control, airway opening with cervical control and immobilization stood out. As for the access to the health system through MPHC, most victims were taken to the referral hospital in trauma emergency care, demonstrating that care is mainly concentrated in the SUS network and that the zero vacancy principle prevails.

Inpatient care needs to be redistributed through the Regulation Central, with a view to impeding the overcrowding of large and medium-sized hospital, causing congestion of the ambulance flow and consequent difficulties to obtain hospital beds due to the high occupancy rates and the long hospitalization time of the victims.
